# Stimuli-Responsive Oligourea Molecular Films

**DOI:** 10.1021/acsami.4c04767

**Published:** 2024-06-07

**Authors:** Arkadiusz Grempka, Damian Dziubak, Anna K. Puszko, Paulina Bachurska-Szpala, Maxim Ivanov, Paula M. Vilarinho, Karolina Pulka-Ziach, Slawomir Sek

**Affiliations:** † Biological and Chemical Research Centre, Faculty of Chemistry, University of Warsaw, Zwirki i Wigury 101, Warsaw 02-089, Poland; ‡ Faculty of Chemistry, University of Warsaw, Pasteura 1, Warsaw 02-093, Poland; § Department of Materials and Ceramic Engineering & CICECO—Aveiro Institute of Materials, University of Aveiro, 3810-193 Aveiro, Portugal

**Keywords:** foldamer, peptidomimetics, oligourea, monolayers, electric field, dipole moment

## Abstract

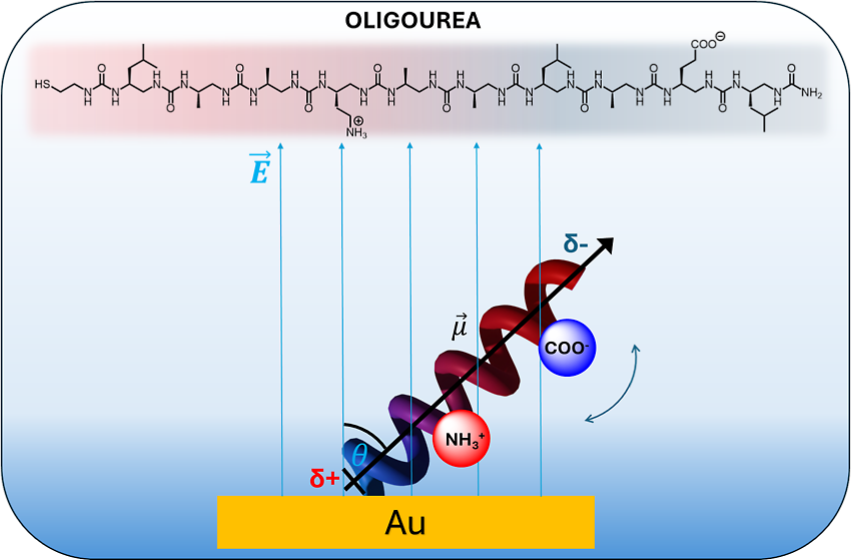

We have designed and synthesized a helical cysteamine-terminated oligourea foldamer composed of ten urea residues featuring side carboxyl and amine groups. The carboxyl group is located in proximity to the C-terminus of the oligourea and hence at the negative pole of the helix dipole. The amine group is located close to the N-terminus and hence at the positive pole of the helix dipole. Beyond the already remarkable dipole moment inherent in oligourea 2.5 helices, the incorporation of additional charges originating from the carboxylic and amine groups is supposed to impact the overall charge distribution along the molecule. These molecules were self-assembled into monolayers on a gold substrate, allowing us to investigate the influence of an electric field on these polar helices. By applying surface-enhanced infrared reflection–absorption spectroscopy, we proved that molecules within the monolayers tend to reorient themselves more vertically when a negative bias is applied to the surface. It was also found that surface-confined oligourea molecules affected by the external electric field tend to rearrange the electron density at urea groups, leading to the stabilization of the resonance structure with charge transfer character. The presence of the external electric field also affected the nanomechanical properties of the oligourea films, suggesting that molecules also tend to reorient in the ambient environment without an electrolyte solution. Under the same conditions, the helical oligourea displayed a robust piezoresponse, particularly noteworthy given the slender thickness of the monolayer, which measured approximately 1.2 nm. This observation demonstrates that thin molecular films composed of oligoureas may exhibit stimulus–responsive properties. This, in turn, may be used in nanotechnology systems as actuators or functional films, enabling precise control of their thickness in the range of even fractions of nanometers.

## Introduction

Stimuli-responsive surfaces, so-called “smart” surfaces, have become the subject of intense research over the past decades due to their potential use in technologies such as electronic and spintronic devices, cell culture, tissue engineering, biosensors, high-tech, drug delivery, and regenerative medicine.^1−5^ The smart surface is the surface functionalized by molecular layers that possess two parts: an anchoring part for bonding to the gold or silica substrates (usually a thiol or silane derivatives)^6^ and a functional group that provides a quantity response to external stimuli. In this context, self-assembled monolayers (SAMs) of thiol derivatives are commonly employed to provide covalently bonded molecular films. SAMs find application in catalysis,^7−9^ biochemical and chemical sensing,^10−13^ molecular electronics,^14−19^ nanolubrication,^20^ and protection from corrosion.^21,22^ The functional part of the “smart” surfaces can be fabricated using various compounds that exhibit desired structural changes induced by external factors such as temperature, pH, or the presence of an external electric field. Due to the enormous structural diversity and the possibility of designing molecules for specific purposes, stimuli-responsive materials and surface modifications based on peptides are very popular.^23−26^ Unfortunately, a significant disadvantage of short peptides is the poorly defined secondary structure and its dependence on the amino acid sequence, as well as the preservation of the structure after immobilization of peptides on the substrate surface. To overcome this problem, artificial biomimicking oligomers, called “foldamers”, characterized by well-defined and stable secondary structures, were developed.^27^ One of the classes of foldamers are N,N′-linked oligoureas. In oligourea foldamers, the specific arrangement of urea units and additional NH groups in comparison to peptides leads to the formation of 3-centered hydrogen bonds. This unique hydrogen bonding pattern plays a crucial role in the conformational stability of oligoureas, which folds into 2.5-helices with the main parameters similar to peptide α-helices.^28^ This similarity makes oligoureas great structural mimetics of peptides. Moreover, certain side groups, like amine or carboxyl acid, can be introduced to the oligourea helix without disrupting its structure.^35^ In our previous studies, we have used oligourea foldamers as electron transfer mediators and have shown that the mechanism of that process was connected with the length of the oligomer, hence the thickness of the SAM.^29,30^ Further, we have explored the stimuli–responsive properties of oligourea thin films immobilized on gold.^31,32^ We have found that the potential-dependent changes in the orientation of oligourea molecules resulted from the presence (and the direction) of the significant dipole moment of oligourea helices and, for electroactive oligoureas with a ferrocene moiety, also by the presence of a positive charge of the redox site generated upon oxidation.

In this paper, we present a study on self-assembled monolayers of oligourea consisting of 10 urea residues and cysteamine termination with side carboxyl and amine moieties (**OU-10u**) located close to the negative and positive poles of the helix dipole, respectively. We have explored the effect of an electric field on the SAMs in a solution using electrochemical surface-enhanced infrared absorption spectroscopy (EC-SEIRAS), as well as in the air using atomic force microscopy (AFM) methods that provide mechanical and electrochemical measurements at the nanoscale level. In both conditions (in air and the buffer of neutral pD), the helices of oligoureas exist as zwitterions; thus, we could observe an enhancement of the intrinsic dipole moment due to the charged side moieties and their response to the external stimuli (electric field).

## Experimental Section

### Synthesis of Oligourea with Side Carboxylic and Amine Groups (**OU-10u**)

Oligourea was synthesized on 200 mg NovaPeg Rink amide resin under microwave irradiation using succinimidyl (2-azidoethyl)carbamate derivatives as building blocks (N_3_-BB), following previously reported procedures.^33^ The syntheses of building blocks, derivatives of Ala, Leu, Glu, Dab, and cysteamine, were already published.^29,33−35^ Coupling and azide reduction steps were monitored by the chloranil test. Coupling conditions: activated N_3_-BB building blocks (1.5 equiv relative to the resin loading) were dissolved in DMF (2 mL) in the presence of DIPEA (3 equiv), added to the reaction vessel, and reacted with the resin at 50 W, 60 °C for 30 min. Each coupling step was repeated until the negative result of the chloranil test was observed (two or three times depending on the building block). After completion of the coupling step, the resin was filtered and washed with DMF (4 × 2 mL), and the azide reduction step was performed. Azide reduction conditions: Before the reduction step, the resin was washed with 1,4-dioxane/H_2_O (2 mL, 7:3 v/v). The reaction of the azide reduction was performed with 1 M PMe_3_ solution in THF (10 equiv relative to the resin loading) in a mixture of 1,4-dioxane/H_2_O (2 mL, 7:3 v/v) at 50 W, 60 °C, 30 min. The reduction step was repeated until the positive result of the chloranil test was observed (two or four times depending on the length of the foldamer on the resin). After the reaction, the resin was filtered and washed with 1,4-dioxane/H_2_O (1 × 2 mL) and DMF (4 × 2 mL). As the last residue, Trt-cysteamine succinimidyl carbamate was attached under the same conditions as applied for N_3_-BB. When the synthesis of the compound was finished, the resin was transferred into the syringe with a frit, washed with DCM (5 × 2 mL), Et_2_O (5 × 2 mL), and dried in the desiccator. The compounds were cleaved from the resin with a mixture of 95:2.5:2.5 TFA/TIS/H_2_O (5 mL). The syringe was shaken for 4 h. After that, the resin was filtered and washed with TFA (1 × 2 mL) and ACN (3 × 2 mL). All the filtrates were combined, and the solvents were evaporated to dryness. The crude product was precipitated with cold Et_2_O and purified by semipreparative HPLC using a Jupiter 4u Proteo 90 Å column (10 × 250 mm) at a flow rate of 5 mL/min. The mobile phase was composed of 0.1% (v/v) TFA-H_2_O (solvent A) and 0.1% TFA-MeOH (solvent B). The detection was performed at 200 nm. The used gradient was as follows: 60–90% B in 40 min, then 90% in 6 min (gradient 1). Analytical HPLC characterization was performed on a Jupiter 4u Proteo 90 Å column (4.6 × 250 mm) at a flow rate of 1 mL/min and the detection at 200 nm with the same solvent system as for semipreparative HPLC. The used gradient was as follows: 50–100% B in 33 min and 100% B in 5 min (gradient 2).

#### HS(CH_2_)_2_NHCONH-Leu^u^-Ala^u^-Ala^u^-Dab^u^-Ala^u^-Ala^u^-Leu^u^-Ala^u^-Glu^u^-Leu^u^-NH_2_ (**OU-10u**)

MS: ESI^+^*m*/*z*: 667.9 [M + 2H]^2+^, 1334.8 [M + H]^+^, ESI^–^*m*/*z*: 1332.8 [M – H]^−^, 1446.8 [M + TFA]^−^; *t*_R_ (gradient 2) = 24.4 min. Chromatograms and MS and ECD spectra are shown in Figures S1 and S2 in the Supporting Information.

### Atomic Force Microscopy

Prior to self-assembly, an Au(111) substrate was immersed in piranha solution [3:1 (v/v) concentrated H_2_SO_4_/30% H_2_O_2_] for 24 h, washed clean with ultrapure water, and flame-annealed to obtain a well-defined Au(111) surface. Then, the substrate was immersed in a methanol solution of **OU-10u** at a concentration of 0.1 mg/mL for 24 h, rinsed with methanol, and gently dried with compressed air. The AFM images were recorded in air using Dimension Icon instrument (Bruker) with ScanAsyst-Fluid+ tips (resonance frequency 150 kHz, force constant 0.7 N/m). To measure the thickness of the self-assembled monolayers, AFM-based nanolithography was performed. A few scans with a high exerted force (around 30 nN) were performed on a small area (300 × 300 nm) to remove the monolayer. Subsequently, a scan on a bigger area (e.g., 3 × 3 μm) was performed with a smaller force (around 1 nN). The profiles were analyzed with Gwyddion software, and histograms were plotted to obtain the mean value of thickness. Moreover, by knowing the length of the helices, an approximate tilt angle was calculated. The nanomechanical properties, including Young’s modulus, were acquired using the PeakForce TUNA mode. Half of the image was recorded with a bias voltage −1 V applied to the stage and the other with a bias voltage +1 V. Quantitative data was obtained based on the Derjaguin–Muller–Toporov (DMT) model of mechanical contact. The selection of the DMT model was based on previous literature, where it was employed to evaluate the properties of alkanethiol monolayers on gold. Engelkes and Frisbie particularly argued for the DMT model, emphasizing that it accounts for adhesive forces that vary with the bias applied between the tip and substrate. This implies that adhesion is influenced by the long-range electrostatic interaction between the tip and substrate.^36^

### Piezoresponse Force Microscopy (PFM)

The PFM method was employed to measure the electromechanical behavior of the samples at the nanoscale level. PFM was performed with a microscope manufactured from Ntegra NTMDT. In PFM measurements, AC voltage at a frequency of 50 kHz and an amplitude of 10 V was applied to conducting AFM probes (Multi75E-G from Budget sensors with a resonance frequency of 75 kHz and a force constant of 3 N/m). The monolayers of **OU-10u** were organized onto Arrandee gold-coated substrates. In order to perform the measurements of local hysteresis behavior, the switching spectroscopy PFM method was applied, where a sequence of stepwise DC voltage pulses from −10 to +10 V was applied to the tip when it was placed in a predefined position on a mesh 10 × 10 over an area of 1 μm × 1 μm. The measurements of PFM hysteresis loops (magnitude and phase) were acquired in the “DC off” regime, where the contribution from electrostatic forces is minimized. Additionally, PFM measurements were performed with a DC bias (−2, 0, +2 V) applied to the substrate with deposited self-assembled monolayers of the oligourea. The quantification of effective local out-of-plane piezoelectric coefficients (*d*_33_) was done via normalization of magnitude in the PFM hysteresis loop on interferometer-type displacement measurements acquired at the same place.

### Buffer Preparation

All of the ingredients were commercially available and were used without further purification. The Britton-Robinson buffer was prepared as a solution of 0.04 M boric acid, 0.04 M phosphoric acid, and 0.04 M acetic acid in water and was titrated with sodium hydroxide 0.1 M to the desired pD. For the surface-enhanced infrared reflection–absorption spectroscopy (SEIRAS) measurements, a similar buffer was prepared with D_2_O, D_3_BO_3_, D_3_PO_4_, and CD_3_COOD and titrated with NaOD.

### Electrochemistry

The electrochemical measurements were performed with a CHI 750E bipotentiostat (CH Instruments Inc., Austin, TX). The three-electrode cell was used: standard silver chloride Ag|AgCl|sat.KCl as the reference, a platinum plate as the counter electrode, and a Au(111) disc as the working electrode. Prior to the measurements, the gold electrode was cleaned with piranha solution [3:1 (v/v) concentrated H_2_SO_4_/30% H_2_O_2_] for 24 h, washed with ultrapure water, flame-annealed, and immersed in the solution of oligourea of 0.1 mg/mL for 24 h. In order to see the desorption of the molecules, cyclic voltammetry from 0 to −1.5 V (vs Ag|AgCl|sat.KCl) at a rate of 50 mV/s was performed in a 0.1 M KOH solution as the supporting electrolyte. The potential of zero free charge (pzfc) was measured by chronocoulometry. The potential was set at 600 mV for 6 s, the gold electrode with the self-assembled layers of oligoureas and the bare gold electrode were brought into contact with the supporting solution, and the peak of charge was measured. The procedure was repeated several times with the potential increased by −50 mV until negative peaks were observed. The pzfc was determined by linear regression, and the value is an average of at least 4 measurements.

### Surface-Enhanced Infrared Reflection–Absorption Spectroscopy

**Scheme 1:**
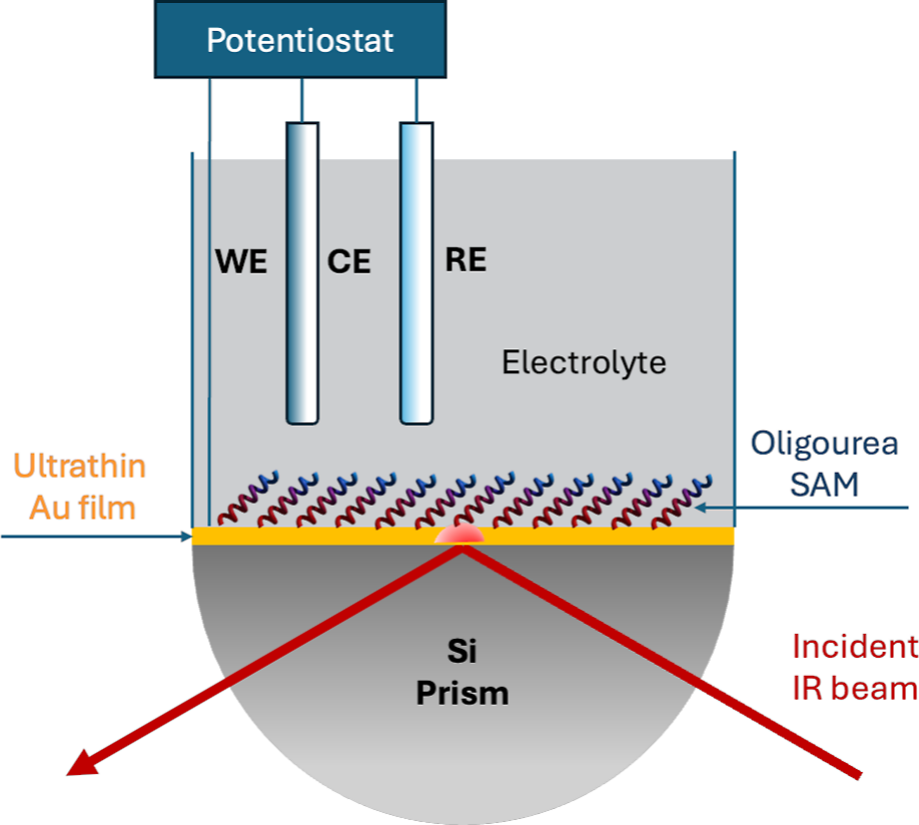
Configuration of the SEIRAS Experimental Setup with Electrochemical Control

The SEIRAS spectra were recorded with a Nicolet iS50 FTIR spectrometer (Thermo Scientific) with a MCT-A detector with a spectral resolution of 4 cm^–1^. A custom-made single reflection accessory in a Kretschmann-ATR configuration was used. This particular configuration involves placing the metal film directly on the planar surface of a silicon hemispherical prism. The incident IR light is directed through the prism, strikes the metal film at an angle greater than the critical angle (usually 60–70°), and excites plasmons at the metal–sample interface. An illustration of the experimental setup is shown in Scheme 1.

The silicon hemisphere prism with a thin layer of gold was prepared by using a previously established protocol.^37^ Self-assembled monolayers of **OU-10u** were created by leaving the so-prepared prism in a solution (0.1 mg in 1 mL of methanol) of oligourea for 24 h to self-assemble. The spectra were recorded under electrochemical control in a three-electrode cell; the counter electrode was a Pt foil; the reference—Ag|AgCl|sat.KCl electrode; and the working was gold wire brought into contact with the thin layer of gold on top of the Si substrate. The spectra were recorded in a potential range from +0.4 to −0.4 V with a step of 0.1 V. The results are presented in absorbance, defined as *A* = −log(*I*/*I*_0_), where *I* are the intensities of IR radiation for the measured potentials and *I*_0_ is the intensity at the potential closest to the pzfc determined from electrochemical measurements. The supporting electrolyte was deuterated Britton–Robinson buffer at pD = 7. The data were treated with OMNIC and Origin software. 2D-COS maps were obtained from the series of differential spectra recorded as a function of the potential applied to the electrode using the software package 2Dshige © Shigeaki Morita, Kwansei-Gakuin University, 2004–2005.

## Results and Discussion

**Figure 1:**
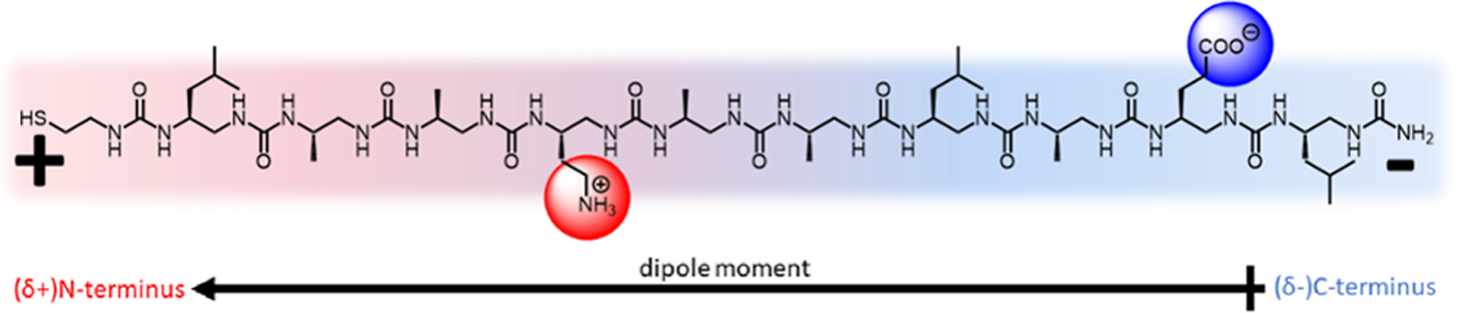
Chemical structure **OU-10u** with the indication of N-terminus and C-terminus, additional charges in the zwitterion form, and poles of the internal dipole moment (red for positive and blue for negative).

Oligourea foldamer was designed to contain the SH group to enable the attachment of the compound to the gold surface. Moreover, residues with amino as well as carboxylic groups, derivatives of Dab and Glu, were incorporated into the helix (see Figure 1). The position of those derivatives was designed to place a Dab-like residue close to the positive pole of the helical dipole and a Glu-like residue close to the negative pole. Furthermore, the sequence of the oligourea foldamer was chosen to place residues with NH_2_ and COOH groups on the same side of the helix, one above the other (with a distance of four residues between them).

The surface concentration of the oligourea molecules, as determined by the reductive desorption in cyclic voltammetry (Figure S3 in the Supporting Information), is (1.8 ± 0.5) × 10^–11^ mol/cm^2^ for **OU-10u**. Based on this value, the average surface area per single molecule can be calculated, which is 9.22 nm^2^. The resulting value of the molecular area indicates a high degree of freedom of movement of the molecules forming the surface film. The mean thickness of the monolayer in air, as determined by AFM, was (1.24 ± 0.21) nm (see Figure S4 in the Supporting Information for the details of the methodology). It is significantly less than the thickness of self-assembled monolayers formed by oligoureas of similar length described previously, which is approximately 2.1 nm.^31^ Due to the smaller packing density, the helical molecules in the SAM tend to be tilted rather than stand vertically on the surface. In fact, we can estimate the angle between the surface normal and the axis of the helix as around 59° ± 6° (see Figure S5 in the Supporting Information).

The measured pzfc was (+0.44 ± 0.03) V for **OU-10u** versus a standard silver chloride Ag|AgCl|sat.KCl as a reference. It is shifted toward more negative potentials compared to the bare gold electrode where the measured pzfc was (+0.53 ± 0.01) V. Since the sulfur–gold bond is created without any significant charge transfer between these two atoms,^38,39^ we can consider the dipole moment of the helices enhanced by the presence of charged side groups to explain the shift.^40^

The direction of the movements of the self-assembled monolayers of oligourea helices was determined by surface-enhanced infrared absorption spectroscopy under electrochemical control (EC-SEIRAS). In a SEIRAS experiment, the metal islands are polarized through the excitation of localized plasmon modes. The dipoles induced within these islands generate a local electromagnetic field that enhances the IR absorption of molecules adsorbed on the metal islands. Notably, the electric field that excites these adsorbed molecules is oriented perpendicular to the island surface at any point. This underpins the surface selection rule, which states that only vibrational modes causing dipole changes perpendicular to the metal surface are SEIRA-active. Consequently, the band intensity varies as a function of the angle between the given transition dipole and the surface plane of the metal.^41^ Hence, the intensity of the signal depends on both the amount of the absorbed molecules and their orientations. Two bands are important in the analysis of the helical oligourea: urea I and urea II, named, by analogy, similarly to amide I and amide II bands present in the α-helix in peptides and proteins. The former is carboxyl C=O stretching present near 1630 cm^–1^. The latter is coupled C–N stretching/N–H deformation present at 1571–1578 cm^–1^. However, due to the interference of H_2_O with the spectral region of interest, the supporting electrolyte, Britton-Robinson buffer, was prepared in D_2_O with all of the ingredients deuterated (D_3_BO_3_, D_3_PO_4_, CD_3_COOD, and NaOD). In such conditions, isotope exchange occurs and N–H becomes N–D, which shifts the urea II band to 1460–1490 cm^–1^.^42,43^ Due to a small contribution of the N–H vibration in the urea I band, in this case, a slight shift of 5–8 cm^–1^ toward lower wave numbers may also be observed. Both C=O and N–H bonds are assumed to be oriented uniformly along the helical axis; however, the oscillation of the dipole moment attributed to the urea I band is roughly parallel to this axis, while the change of the dipole moment related to urea II is more perpendicular to this axis.^44,45^ An increase/a decrease in the urea I/urea II band indicates a change of the orientation of the helices with respect to the gold surface, in that more vertical alignment of the helices to the surface results in an increase in the urea I band and a decrease in the urea II band.

**Figure 2:**
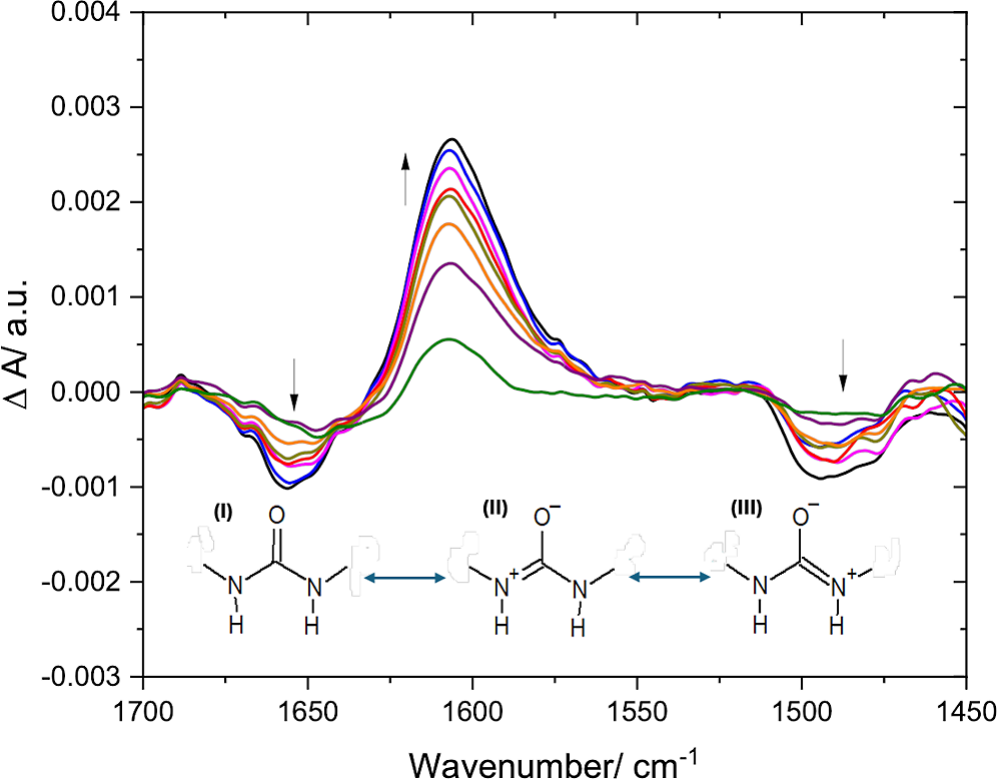
Potential-dependent SEIRA spectra for the **OU-10u** monolayer absorbed on gold. The arrows show the direction of the spectral changes varying upon stepping potential from +0.3 to −0.4 V with a −0.1 V increment. The *I*_0_ spectrum was recorded at a potential of +0.4 V. Inset: Resonance structures of the urea moiety.

**Figure 3:**
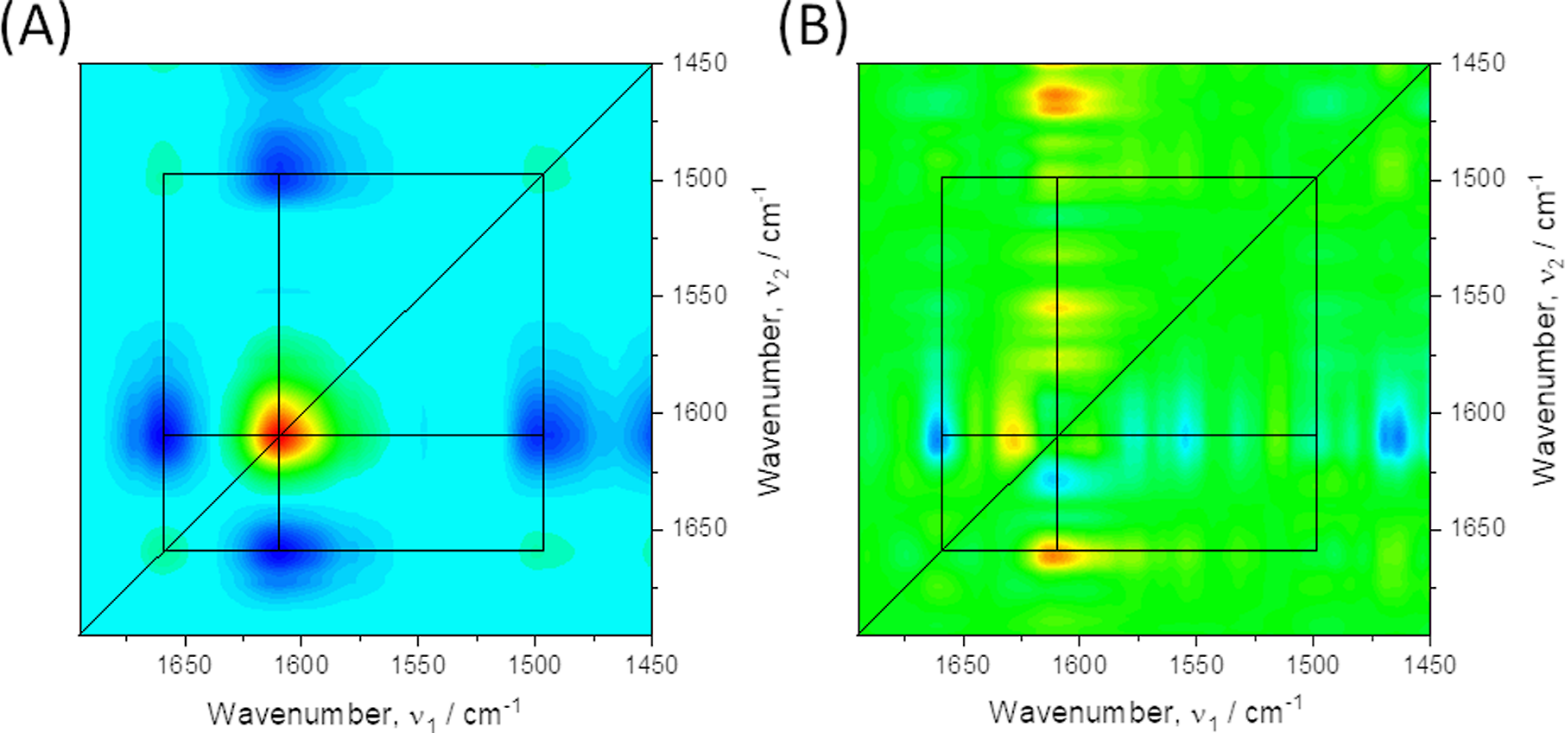
2D-COS analysis of potential-dependent SEIRA spectra between +0.3 and −0.4 V. (A) Synchronous correlation; zero intensity is cyan, blue is negative, and red is positive; (B) asynchronous correlation; zero intensity is green, blue is negative, and red is positive.

The SEIRAS experiments were performed under electrochemical conditions in the potential range between +0.5 and −0.4 V. The response of oligourea molecules (**OU-10u**) was investigated in the deuterated buffer at neutral pD = 7. It is assumed that in the neutral condition (pD ≈ 7), most of the carboxyl and amine groups exist in their charged forms, and the oligourea is a zwitterion. Since at the pzfc we can assume that there are no charges on the electrode and the self-assembled molecules are in their near-equilibrium orientation, the spectrum at the potential closest to the pzfc was used as the reference spectrum *I*_0_ (at +0.4 V for **OU-10u**). The spectra presented in Figure 2 were recorded with a potential step of −0.1 V. The working electrode with a SAM of oligourea became increasingly negatively charged, and an increasingly stronger electric field acted on the helices. In the spectra, we see a noticeable increase of the positive band at ∼1610 cm^–1^ and the development of the negative band at 1488–1495 cm^–1^. These bands were identified as urea I and urea II bands, respectively. Nevertheless, the urea I band is significantly shifted toward lower frequencies compared to the typical value reported for oligourea ∼1630–1640 cm^–1^. The displacement of the urea I band in SEIRA spectra toward lower frequencies for surface-confined long oligourea has been reported in prior literature, a phenomenon attributed to the isotope exchange effect and possibly increased strength of hydrogen bonds.^32^ While this holds true in this investigation, the observed shift in our case is notably more pronounced, underscoring the unique and intensified impact of the increased hydrogen bonding strength in the current experimental system. In the spectra of surface-confined long oligourea, there is also a weak band around 1650–1660 cm^–1^.^32^ However, in our examined compound, this band is significantly more pronounced, particularly as a distinct negative feature at ∼1660 cm^–1^. We believe that these findings strongly indicate that upon adsorption and under the influence of an electric field, there is a substantial amplification in the strength of hydrogen bonding within the oligourea backbone.^46^ Since hydrogen bonding to oxygen stabilizes the resonance structure with charge transfer character, there is an increase in the contribution of the resonance structure (see structures II and III in the inset in Figure 2).^47^ Such an effect might also be amplified by the presence of the charged side groups, which can influence the electron density distribution. Thus, the main chain carbonyl bond decreases its order, becoming more like a single bond, while the carbon–nitrogen bond increases its order, acquiring a double bond character and becoming more imine-like. Such an effect was reported in the literature for urea, and it was demonstrated that it can contribute to the appearance of the IR band at ∼1650 cm^–1^.^48^ The observed spectral changes can also be explained in terms of the Stark effect, where the bands shift or split due to an external electric field. However, it does not exclude the effects of variations in the hydrogen bonding strength. In our case, the electric field likely affects electron densities in amide bonds since the equilibrium between amide resonance structures is known to be sensitive to the electrostatic field. This can alter the hydrogen bonding strength and contribute to the electric-field-induced changes observed in SEIRA spectra. Since the SEIRA spectra obey surface selection rules, it is possible to track dynamic changes in the orientation of the molecules forming the monolayer during stimulation with an electric field. The urea I band recorded during the sequential potential steps is positive, and its intensity grows as the negative charge of the electrode increases. This results from the reorientation of the transition dipole moment related to the stretching vibration of the carbonyl group, which increases its tilt angle with respect to the surface of gold. Hence, the oligourea molecule adopts a more vertical orientation due to the repulsion of the C-terminus from an increasingly negatively charged surface. At the same time, the band at ∼1660 cm^–1^ corresponding to the stretching vibrations from the C=N^+^ bond also increases in absolute intensity, but it is negative. This may indicate a decreasing angle between the transition dipole moment of the C=N^+^ bond and the electrode surface. Therefore, this would also correspond to the situation when the molecule, under the influence of an electric field, takes on an increasingly vertical orientation as the negative charge of the surface increases. The analysis of the urea II band leads to similar conclusions. Since the contribution from N–H bending vibrations dominates in this band, negative intensities indicate a decrease in the angle between the transition dipole moment and the surface of the metal. It means that the carbonyl and N–H bonds become more perpendicular to the surface. To further deepen the spectroscopic analysis, we used the sequence of SEIRA spectra recorded during a perturbation introduced to the system in the form of a varying electric field to construct two-dimensional correlation spectra (2D-COS). The analysis of such spectra makes it possible to trace the correlation between electric field-induced changes in the intensity of the bands.^49,50^Figure 3A,B shows synchronous and asynchronous correlation spectra, respectively, in the potential range of +0.3 to −0.4 V. Synchronous 2D intensity correlation shows spectral changes occurring simultaneously, while asynchronous 2D intensity correlation shows spectral changes occurring sequentially or those that are unsynchronized. Moreover, the asynchronous spectra allow differentiation and deconvolution of overlapping bands.^49^ The synchronous spectrum (Figure 3A) is always symmetrical with respect to the diagonal and shows peaks located on the diagonal called auto peaks, the intensity of which represents the intensity of individual bands. The peaks located symmetrically off the diagonal are called cross peaks, reflect the degree of band correlation, and provide information about the direction of relative changes in the orientation of electric dipole transition moments.

In Figure 3A, the auto peaks on the diagonal are located at ∼1660, ∼1610, and ∼1495 cm^–1^, which can be assigned to the C=N^+^ stretching vibrations and the urea I and urea II bands, respectively. They represent the reorientation of electrical dipole transition moments for individual bonds in the oligourea skeleton caused by the variation of the electric field. Intense cross peaks appearing at off-diagonal positions indicate that the bands’ intensity changes under the influence of the potential applied to the electrode are coherent. However, differences in the sign of cross-peaks are visible. Cross-peaks at 1660 and 1610 cm^–1^ are negative, which means that the reorientation of individual groups C=N^+^ and C–O^–^ is synchronized, but it occurs in opposite directions—the electric dipole transition moment angle with respect to the metal surface decreases or increases, respectively.^51^ A similar relationship arises from the analysis of negative cross peaks at 1495 and 1610 cm^–1^. Therefore, an increase in the tilt angle between the electric dipole transition moment and the carbonyl stretching vibration is accompanied by a decrease in the tilt angle for the N–H bending vibration. At the same time, we observe positive cross peaks at 1660 and 1595 cm^–1^, which prove that the reorientation of electric dipole transition moments for these vibrations occurs in the same direction. Therefore, this confirms the previous conclusion that as the negative charge of the electrode increases, the oligourea molecules assume an increasingly vertical orientation. Further analysis was based on the asynchronous correlation shown in Figure 3B. In this case, the sign of the asynchronous correlation provides information about the temporal relationship between intensity fluctuations. A positive sign means that the change in intensity at ν_1_ occurs earlier than that at ν_2_. In turn, a negative sign means that the change in intensity at ν_1_ occurs later than at ν_2_. This relationship reverses when the corresponding synchronous correlation is negative.^49,50^ As can be seen in the asynchronous 2D correlation spectrum, the bands at 1660 and 1495 cm^–1^ do not show an asynchronous correlation, which means that the changes in the intensity of these two bands are completely synchronized. However, the positive asynchronous correlation at 1610/1660 cm^–1^ and the negative synchronous correlation for the same pair lead to the conclusion that the reorientation of the transition dipole moment of C=N^+^ bond stretching precedes the reorientation of the transition dipole moment of carbonyl bond stretching. The analysis of 1495/1610 cm^–1^ asynchronous correlation leads to the conclusion that the reorientation of the transition dipole moment resulting from N–H bond deformation vibrations also precedes the reorientation of the transition dipole moment of the carbonyl group, which is understandable due to the previously mentioned complete synchronization of the 1660 and 1495 cm^–1^ bands. Taking into account that the perturbation of the system involves changing the potential applied to the electrode, it can be concluded that the orientation of the electric dipole transition moments related to C=N^+^ stretching and N–H deformation vibrations are affected at less negative potentials than the carbonyl group.^41^ An interesting observation lies in the 2D asynchronous correlation spectrum, which unveils a series of additional cross peaks. They reflect several overlapping bands that change their intensity with varying potential and, at the same time, are not highlighted in the 1D spectrum. Particularly noteworthy are the cross peaks around ∼1610/1630 cm^–1^, indicating some contribution from the intrinsic structure of oligourea closer to its equilibrium distribution of resonance structures. Additionally, some weak cross peaks at 1550/1610 and 1580/1610 cm^–1^ are discernible, potentially stemming from asymmetric stretching vibrations of charged carboxyl groups within Glu^u^ side chains and deformational vibrations of N–H bonds, wherein no isotopic exchange has occurred.

**Figure 4:**
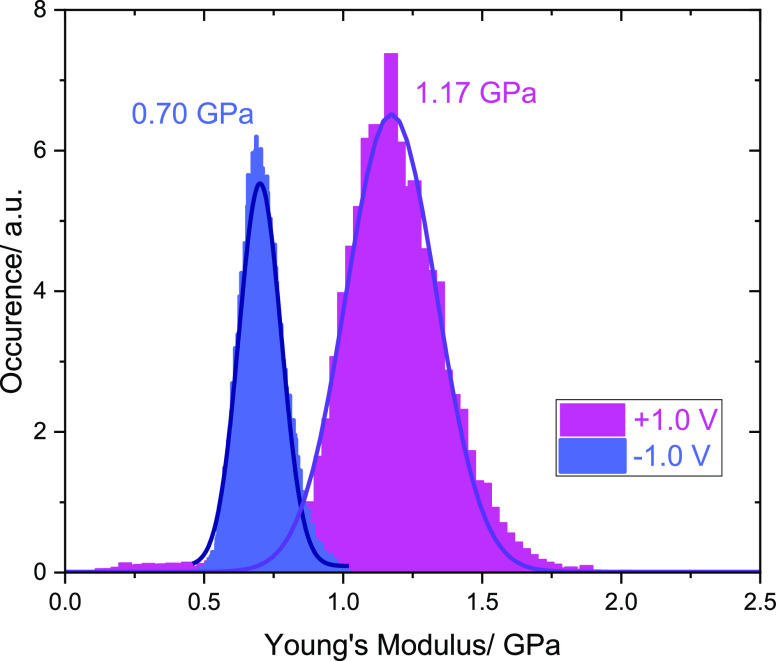
Histograms obtained from the nanomechanical mapping of Young’s modulus of the oligourea monolayer at +1.0 and −1.0 V bias voltages applied between the AFM tip and the substrate.

Finally, the response of the oligourea monolayers to an external electric field was also examined in air. Initially, the nanomechanical properties of the monolayer were evaluated by determining Young’s modulus after polarization of the substrate to +1.0 and −1.0 V, respectively. The results are presented in the form of histograms in Figure 4. The obtained values of Young’s modulus are 1.17 ± 0.19 and 0.70 ± 0.09 GPa, which are comparable to those reported for the Langmuir–Blodgett films and thiol derivative monolayers on gold.^52,53^

It can be seen that with positive polarization of the substrate, Young’s modulus is significantly higher (hence the layer is stiffer) compared to negative polarization. This effect can be interpreted based on changes in the orientation of the molecules caused by variations in the electric field generated between the substrate and the AFM probe. In other words, in the case of positive polarization, the molecules decrease their tilt angle relative to the metal surface, which results from the electrostatic attraction of the negative pole of the helix and the negatively charged carboxyl group toward the positively charged surface. Thus, the monolayer globally decreases its thickness and the molecules have less space to respond to the load applied by tilting, which results in increased stiffness and an increase in the Young’s modulus value. In this case, the effect of the underlying substrate on the measured stiffness will also be better pronounced. In the opposite situation, with negative polarization of the substrate, the negative pole of the helix dipole (as well as the negatively charged carboxyl group) is repelled from the negatively charged surface, which increases the thickness of the monolayer, and consequently, molecules become more flexible and can respond to the load applied by tilting to some extent, resulting in a decrease in the value of Young’s modulus. Another important factor to consider in the specific case of the oligourea described in our study is the possibility of forming salt bridges between charged side groups–carboxyl and amino. The formation of such bridges could potentially occur at large tilts of the helix and thus with a positively charged substrate. Their presence would certainly stiffen the structure of the monolayer, contributing to an increase in the value of Young’s modulus. With a negatively charged substrate, the helices are more vertically oriented, which prevents the formation of bridges due to the relatively large distance between the adsorbed molecules and the charged groups. Moreover, in the case of a more vertical orientation of the helix, it generates more free space between the molecules than in the case of significant tilting. This would also explain the higher value of Young’s modulus in the case of more steeply tilted oligourea molecules. In general, this behavior of the molecules forming the monolayer is in agreement with the results obtained by using EC-SEIRAS, where the reorientation of molecules in the electric field was directly observed.

**Figure 5:**
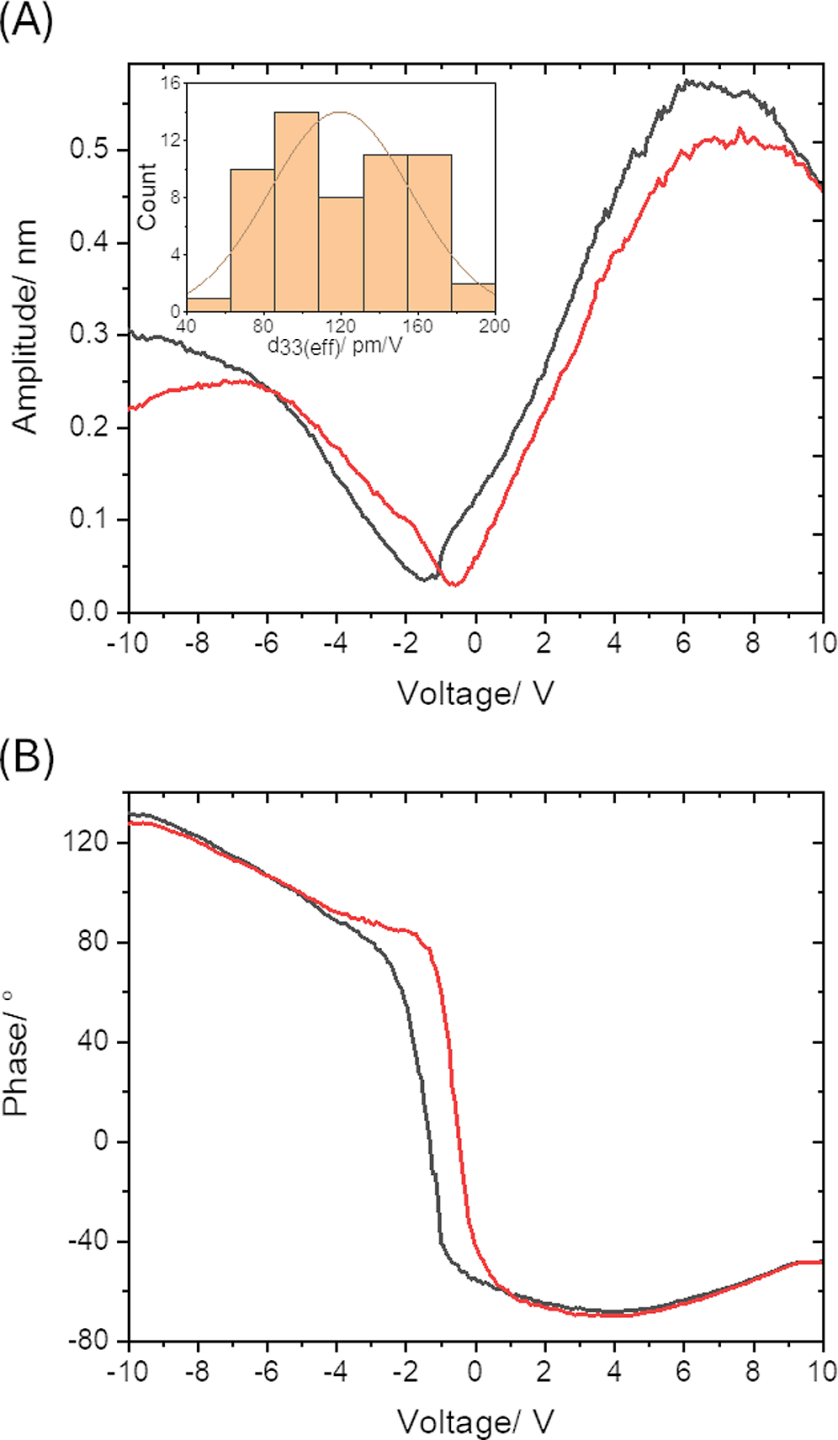
PFM hysteresis loops, amplitude (A), and phase components (B) for monolayers of **OU-10u** molecules with a bias of −2 V applied to the gold substrate. The inset: a histogram of the *d*_33_(eff) coefficient obtained from linear fits around 0 for 100 curves.

Given that oligourea molecules seem to align in the presence of an electric field, we investigated the local electromechanical properties of the molecular films by PFM. A sequence of DC voltage pulses from −10 to +10 V and then back to −10 V was applied to the tip. For each sample, 100 loops were acquired in a mesh 10 × 10 over an area 1 × 1 μm. Additionally, a bias was applied to the substrate: first 0 V, then +2 V, and then −2 V. Representative piezo loops are shown in Figure 5 at the applied DC bias voltage of −2 V. The corresponding hysteresis behavior can be seen as a loop for the phase–voltage chart and in the butterfly-like amplitude vs voltage chart where the voltage separation between the minima of the curves in the positive and negative scan directions is the voltage that causes the polarization of the layers to disappear. In order to quantify the dimensional change when an oligourea film is subjected to an electric field, we have also determined the effective local out-of-plane piezoelectric coefficient *d*_33_. The piezo loops for DC bias voltages of 0 and +2 V and the mean values of the *d*_33_ coefficients are shown in Supporting Information in Figures S8–S10. The fitted *d*_33_ coefficient of **OU-10u** was found to be 192 ± 55 pm/V for the monolayers under the DC bias of 0 V applied and 79 ± 21 and 119 ± 36 pm/V for DC bias +2 and −2 V, respectively. It means that the monolayers of **OU-10u** exhibit a surprisingly high piezoelectric response, and the values of *d*_33_ demonstrate that the effect is strongest when the molecules are in the most neutral state without DC bias applied.

## Conclusions

In conclusion, we have successfully designed and synthesized helical cysteamine-terminated oligourea foldamers consisting of ten urea residues with side carboxyl and amine groups. The strategic positioning of these functional groups results in a molecule with a distinct dipole moment, with the carboxyl group situated near the C-terminus contributing to the negative pole and the amine group near the N-terminus contributing to the positive pole. Incorporating these additional charges into the oligourea structure enhances the overall charge distribution along the molecule. Through self-assembly onto a gold substrate and subsequent investigation using SEIRAS, we observed that the orientation of these polar helices within monolayers can be manipulated by applying an external electric field, particularly causing them to reorient more vertically under a negative bias. Additionally, the presence of the electric field induces the rearrangement of the electron density at urea groups, stabilizing resonance structures with charge transfer character. Furthermore, the nanomechanical properties of the oligourea films are affected by the external electric field, suggesting a tendency for molecules to reorient, even in the absence of an electrolyte solution. Remarkably, despite their slender thickness of approximately 1.2 nm, the helical oligourea films exhibit a robust piezoresponse under the same conditions. This observation underscores the potential of thin molecular films composed of oligoureas to serve as stimuli-responsive materials for nanotechnology applications, offering opportunities for their use as actuators or functional films and enabling precise control of the thickness at the nanometer scale.

## Supporting Information Available

Chromatogram, MS (ESI-TOF), ECD spectra, cyclic voltammogram, AFM image, schematic representation of the oligourea helix, ATR spectra, SEIRAS absorbance of the urea I band, PFM hysteresis loops, and e mean values of the d_33_(eff) coefficient (PDF)
