# Supplementary material for: Stimuli-Responsive Oligourea Molecular Films
Source: ACS Appl Mater Interfaces. 2024 Jun 7;16(24):31817–25. doi: 10.1021/acsami.4c04767 (PMC11194770; doi:10.1021/acsami.4c04767)
Supplement: Supplementary file 1 — am4c04767_si_001.pdf [file am4c04767_si_001.pdf]

Supplementary information

Stimuli-Responsive Oligourea Molecular Films

Arkadiusz Grempka[1], Damian Dziubak[1], Anna K. Puszko[2,] Paulina Bachurska-Szpala[2], Maxim Ivanov[3], Paula M. Vilarinho[3], Karolina Pulka-Ziach[2], Slawomir Sek[1*]*

[1] *Biological and Chemical Research Centre, Faculty of Chemistry, University of Warsaw, Zwirki i Wigury 101, Warsaw 02-089, Poland*

[2] *Faculty of Chemistry, University of Warsaw, Pasteura 1, Warsaw 02-093, Poland*

[3] *Department of Materials and Ceramic Engineering & CICECO— Aveiro Institute of Materials, University of Aveiro, 3810-193 Aveiro, Portugal*

*Corresponding authors: karola@chem.uw.edu.pl (K.P.-Z.); slasek@chem.uw.edu.pl (S.S.)

a)

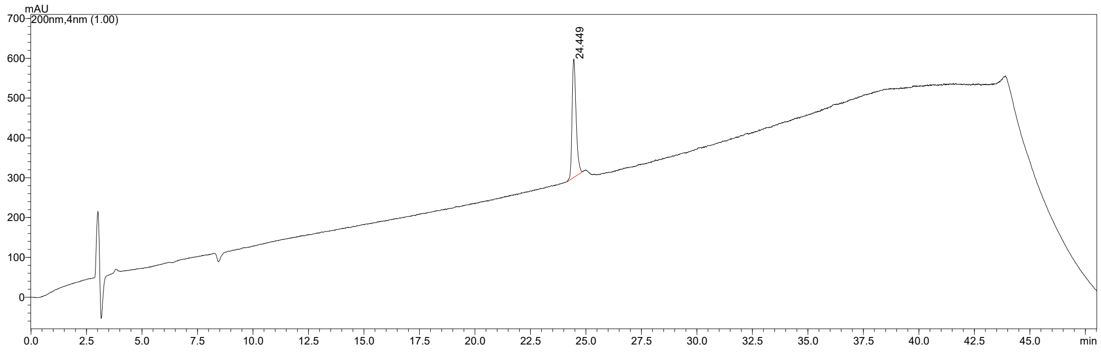

b)

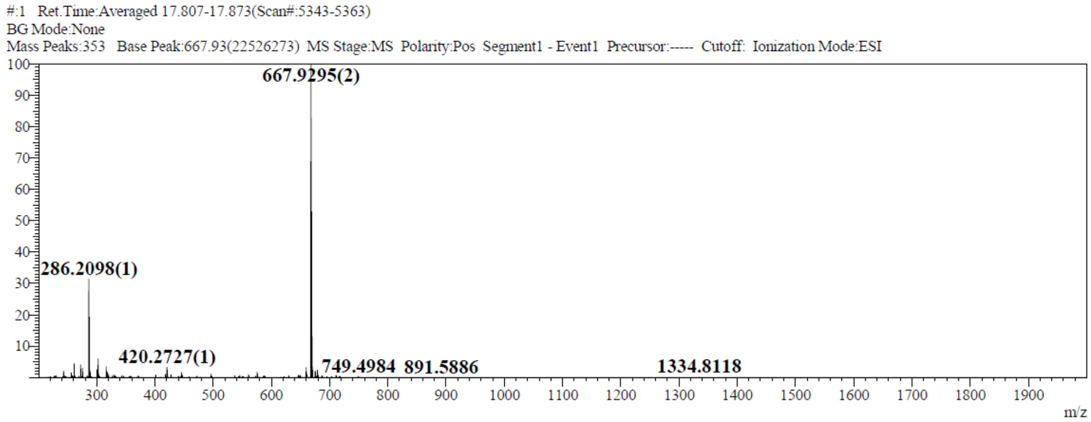

c)

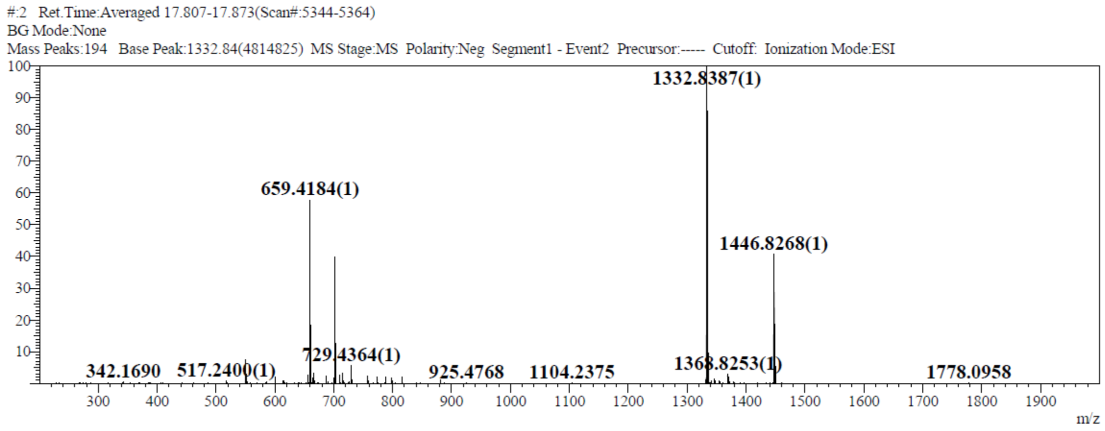

Figure S1. a) Chromatogram of OU-10u (λ=200 nm); b) MS (ESI-TOF) in positive ion mode; c) MS (ESI-TOF) in negative ion mode

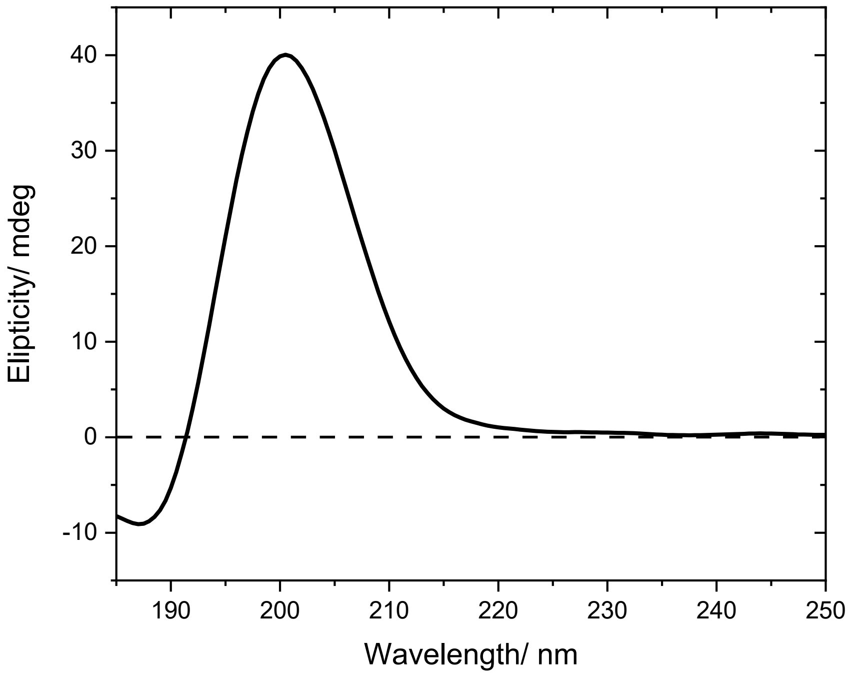

Figure S2. ECD spectra of OU-10u recorded in D_2O exhibiting a strong positive band at λ ~202 nm, which confirms that oligourea adopts 2.5-helical conformation.

In Figure3 we can see a desorption peak of OU-10u absorbed on a gold electrode. By integrating the peak and dividing by Faraday constant and by the surface of the electrode (0.09 cm^2) we obtain the number of molecules per surface unit.

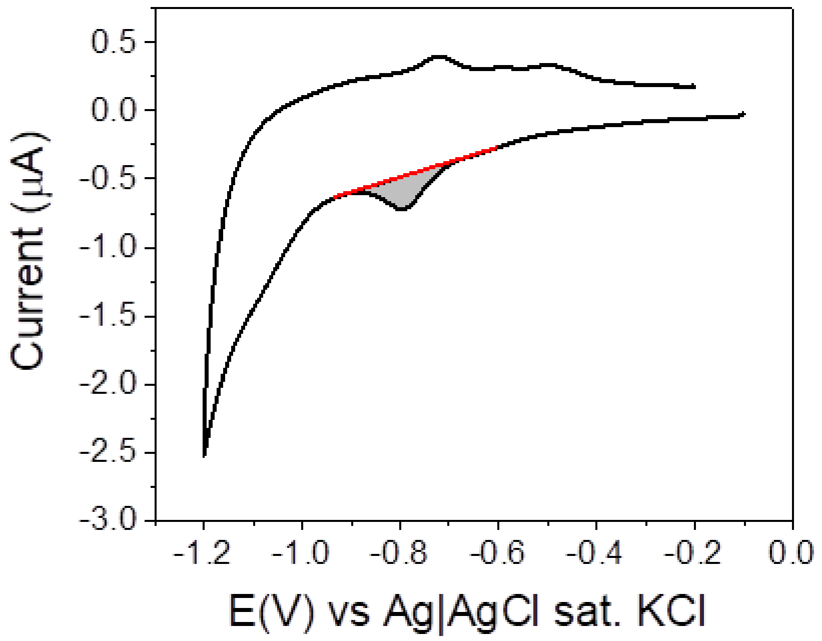

Figure S3. Cyclic voltammogramm. The shadowed area represents desorption on the basis of which surface density is estimated.

In Figure S4 nanolitography performed by Atomic Force Microscopy in air is presented. For each sample at least 3 different scratches were made and the value of thickness is the average of the height of at least 25 profiles.

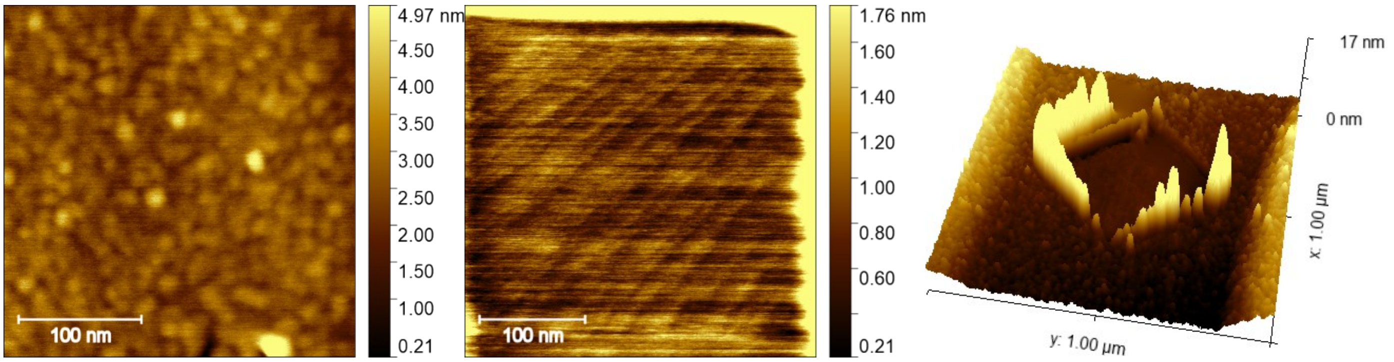

Figure S4. a) An AFM image of the topology of the sample when the applied force was around 800 pN. This is a monolayer of OU-10u. b) An AFM image obtained during scratching of the surface when the applied force was 20nN. The effect of scratching and smoothing the surface can be seen. c) An AFM image of the scratch made with the tip exerting force of 1nN. This scratch lies in a flat region between higher terraces of gold.

Taking into account the parameters of an oligourea helix[1] we can estimate the length of the oligoureas composed of 11 urea residues that folded into a helix:

$$\boldsymbol{length} = \left(\mathbf{10}\,\boldsymbol{residues} \cdot \frac{\mathbf{1}\,\boldsymbol{turn}}{\mathbf{2.48}\,\boldsymbol{residues}} \cdot \frac{\mathbf{5.03\text{Å}}}{\mathbf{1}\,\boldsymbol{turn}}\right) + \mathbf{4.0}\,\text{Å} = \mathbf{24.3\text{Å}}$$

However, the helices are not aligned along the surface normal, but are inclined by a certain angle to the surface normal (see Figure S5). By comparing the calculated length and the thickness of the layers measured by the AFM we can estimate the angle θ between the surface normal and the axis of the helix:

$$\mathbf{OU\text{-}10u:}\ \boldsymbol{\theta} = \mathbf{arccos}\left(\frac{12.4}{24.3}\right) = \mathbf{59.3° \pm 5.8°}$$

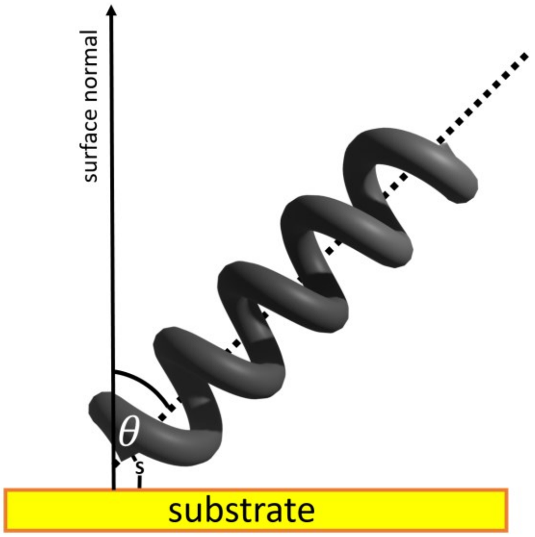

Figure S5. A schematic representation of the oligourea helix tilted by the θ with the respect to the surface normal.

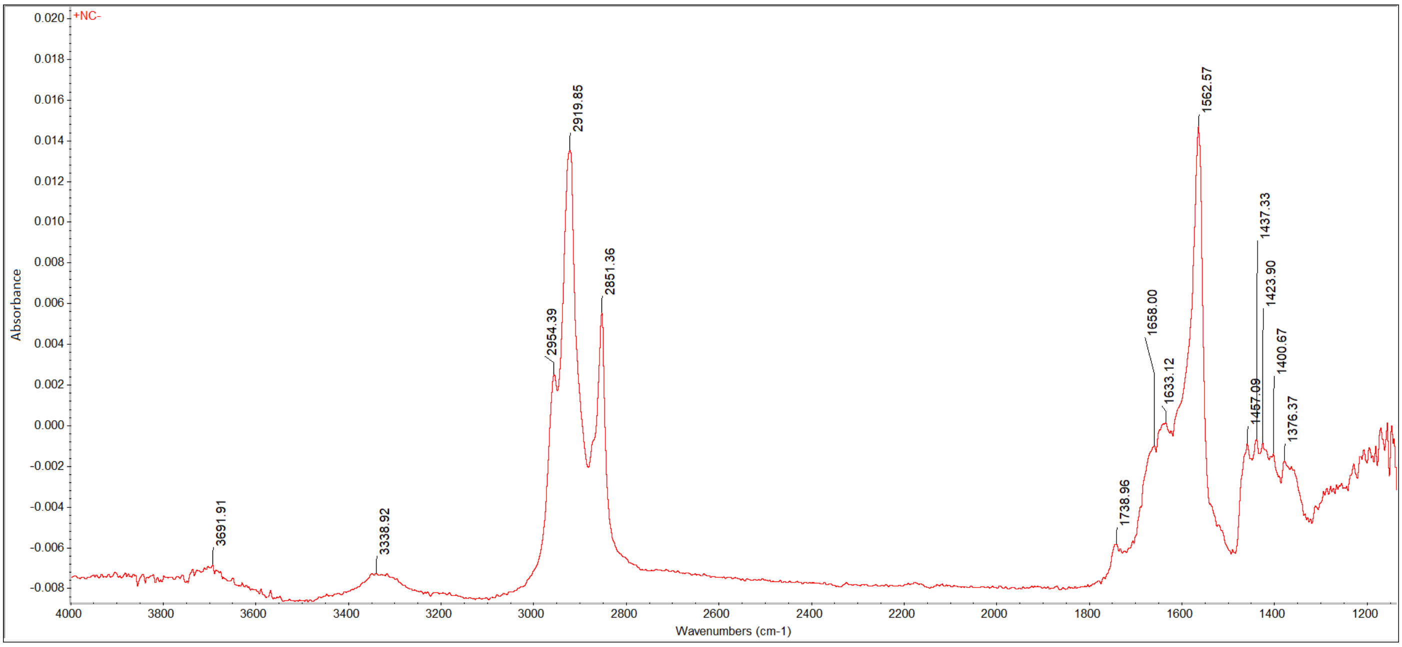

Figure S6. An ATR spectrum of a OU-10u compound deposited from a methanol solution on a silica prism and dried off.

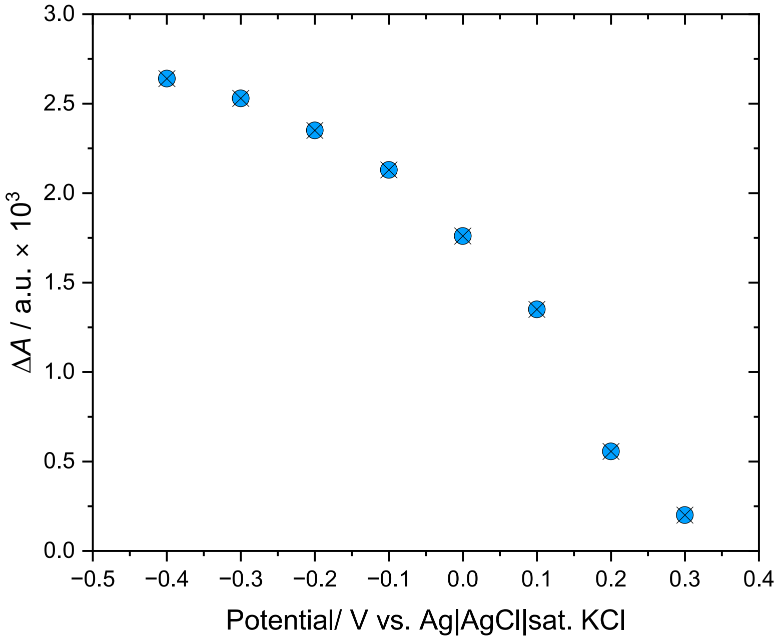

Figure S7. SEIRAS absorbance of urea I band as a function of the potential applied to the electrode modified with OU-10u monolayer.

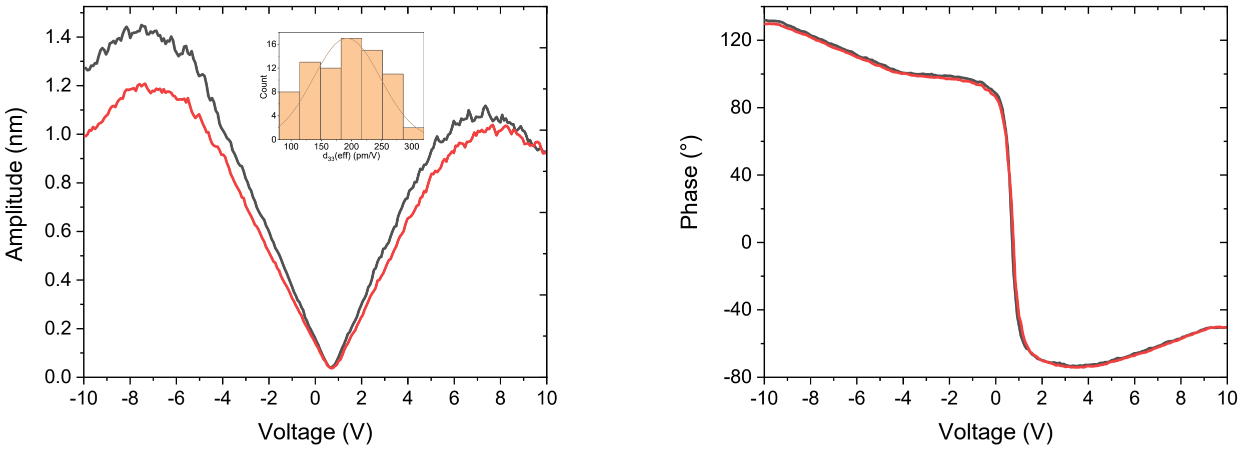

Figure S8. PFM hysteresis loops (amplitude and phase components) for monolayers of OU-10u molecules with a bias of 0 V applied to the gold substrate. The insert: a histogram of the d_{33}(eff) coefficient obtained from linear fits around 0 for 100 curves.

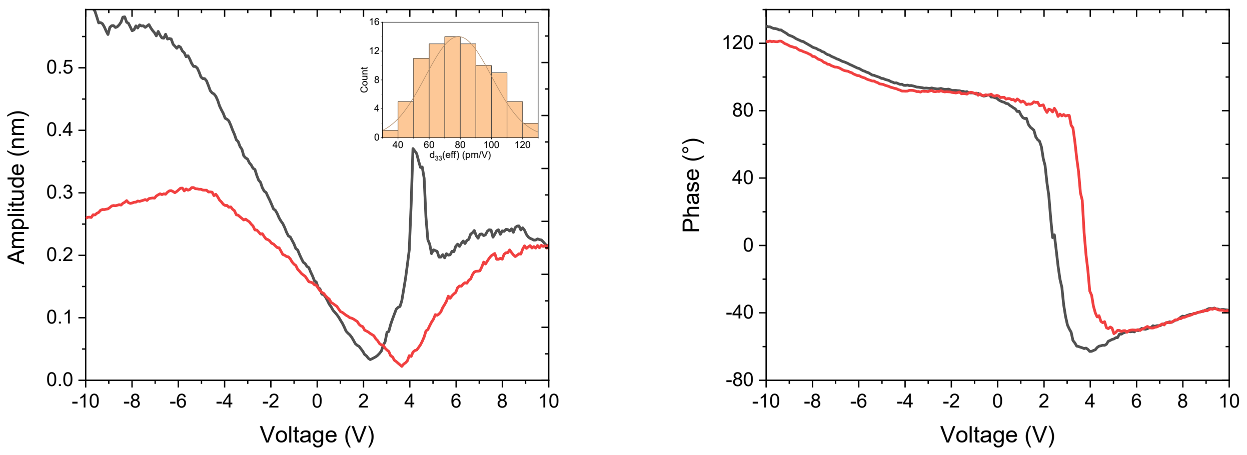

Figure S9. PFM hysteresis loops (amplitude and phase components) for monolayers of OU-10u molecules with a bias of +2V applied to the gold substrate. The insert: a histogram of the d_{33}(eff) coefficient obtained from linear fits around 0 for 100 curves.

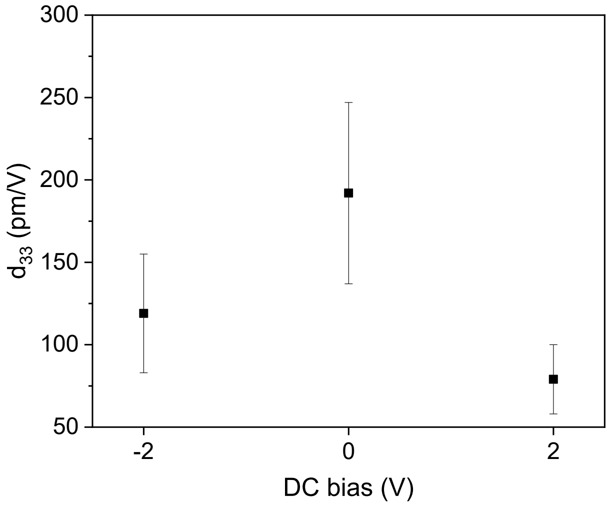

Figure S10. The mean values of d_{33}(eff) coefficient of OU-10u at three different DC biases applied to the gold substrate.

(1) Pulka-Ziach, K.; Sęk, S. α-Helicomimetic Foldamers as Electron Transfer Mediators. *Nanoscale* **2017**, *9* (39), 14913–14920. https://doi.org/10.1039/C7NR05209J
